# Clinical, pharmacokinetic and pharmacodynamic evaluations of metronomic UFT and cyclophosphamide plus celecoxib in patients with advanced refractory gastrointestinal cancers

**DOI:** 10.1007/s10456-012-9260-6

**Published:** 2012-03-02

**Authors:** Giacomo Allegrini, Teresa Di Desidero, Maria Teresa Barletta, Anna Fioravanti, Paola Orlandi, Bastianina Canu, Silvio Chericoni, Fotios Loupakis, Antonello Di Paolo, Gianluca Masi, Andrea Fontana, Sara Lucchesi, Giada Arrighi, Mario Giusiani, Andrea Ciarlo, Giovanni Brandi, Romano Danesi, Robert S. Kerbel, Alfredo Falcone, Guido Bocci

**Affiliations:** 1 Division of Medical Oncology, Azienda USL 5 Pisa, Pontedera, Pisa, Italy; 2 Division of Pharmacology, Department of Internal Medicine, University of Pisa, Via Roma, 55, 56126 Pisa, Italy; 3 Department of Oncology, Transplants and New Technologies in Medicine, University of Pisa, Pisa, Italy; 4 Division of Legal Medicine, Department of Neuroscience, University of Pisa, Pisa, Italy; 5 U.O. Oncologia Medica Az-USL4, Prato, Italy; 6 Dipartimento di Ematologia e Scienze Oncologiche L.A. Seragnoli, Università di Bologna, Policlinico S. Orsola, Bologna, Italy; 7 Sunnybrook Research Institute, Molecular and Cellular Biology Research, University of Toronto, Toronto, Canada

**Keywords:** Metronomic chemotherapy, Gastrointestinal cancer patients, Angiogenesis, Pharmacodynamic biomarkers, Pharmacokinetics, UFT, Cyclophosphamide, GHB

## Abstract

**Aims:**

To evaluate UFT and cyclophosphamide (CTX) based metronomic chemotherapy plus celecoxib (CXB) for the treatment of patients with heavily pre-treated advanced gastrointestinal malignancies.

**Methods:**

Thirty-eight patients received 500 mg/mq^2^ CTX i.v bolus on day 1 and, from day 2, 50 mg/day CTX p.o. plus 100 mg/twice a day UFT p.o. and 200 mg/twice a day CXB p.o. Tegafur, 5-FU, 5-FUH_2_, GHB and uracil pharmacokinetics were assessed. Plasma vascular endothelial growth factor (VEGF), soluble VE-cadherin (sVE-C) and thrombospondin-1 (TSP-1) levels were detected by ELISA and real-time PCR of CD133 gene expression on peripheral blood mononuclear cell was also performed.

**Results:**

Seventeen patients (45%) obtained stable disease (SD) with a median duration of 5.8 ms (range, 4.2–7.4). Median progression free survival (PFS) and overall survival (OS) were 2.7 ms (95% CI, 1.6–3.9 ms) and 7.1 ms (95% CI, 4.3–9.9 ms), respectively. No toxicities of grade >1 were observed. Pharmacokinetics of 27 patients (13/14, SD/progressive disease, PD) after the first treatment of UFT revealed that 5-FU AUC and C_max_ values greater than 1.313 h × μg/ml and 0.501 μg/ml, respectively, were statistically correlated with stabilization of disease and prolonged PFS/OS. VEGF and sVE-C plasma levels were greater in the PD group when compared to SD group. CD133 expression increased only in the PD patients.

**Conclusion:**

Metronomic UFT and CTX with CXB in heavily pre-treated gastrointestinal patients were well tolerated and associated with interesting activity. Potential predictive pharmacokinetic parameters and pharmacodynamic biomarkers have been found.

## Introduction

Interest in metronomic chemotherapy is rapidly growing among both basic researchers and clinical oncologists, especially because of its efficacy in palliative care, low toxicity profile [1] and low cost when using off patent drugs [2]. However, rational strategies for developing new metronomic chemotherapy protocols and schedules are needed in order to improve the knowledge and application of this therapeutic regimen. The antitumor effects of metronomic chemotherapy can be achieved through several mechanisms, including inhibition of angiogenesis and vasculogenesis, blockade of circulating endothelial progenitor cells (CEPs) [3], suppression of HIF-1α expression [4, 5] and, depending on the administered drug and tumor cells being treated, cytotoxic action on tumor cells and stimulation of the immune system [6]. Metronomic chemotherapy has also been proposed as a promising approach to treat patients resistant to standard chemotherapies [7, 8]. Metronomic administration of cyclophosphamide (CTX) in combination with antiangiogenic drugs has shown a potent preclinical activity [9]. Indeed, phase II studies evaluating the impact of metronomic CTX in combination with celecoxib (CXB), a selective cyclooxygenase-2 inhibitor, showed promising antitumor activity [10]. Moreover, celecoxib has been shown to inhibit tumor angiogenesis [11] in preclinical studies, and to induce apoptosis of endothelial cells in tumors of the gastrointestinal tract [12].

UFT, a combination of tegafur, a prodrug of 5-fluorouracil (5-FU) and uracil, has demonstrated clinical anti-tumor activity in many malignancies and, in particular, for the treatment of gastrointestinal cancers [13–15]. It has been successfully tested using metronomic-like protocols in randomized phase III adjuvant therapy trials of non small cell lung cancer [16] and breast cancer [17] where the drug has taken orally every day for 2 years with no breaks. Furthermore, gamma-hydroxybutyric acid (GHB), a metabolite of UFT, has shown antiangiogenic activity in preclinical studies [18]. The rationale of a metronomic chemotherapy strategy based on the combination of UFT and CTX derives in part from their synergistic antitumor activity in mouse models of advanced metastatic disease [19] and also because of evidence that CTX may alter the expression of enzymes such as thymidylate synthase (TS) and dihydropyrimidine dehydrogenase (DPD) in tumor cells in such a way as to render them more sensitive to 5-FU [20]. Given these considerations, we planned a phase II clinical study to evaluate the feasibility and the activity of a regimen combining metronomic UFT plus CTX and CXB in patients with advanced metastatic gastrointestinal cancers, mainly metastatic colorectal carcinoma (mCRC), who failed standard therapies with an acceptable life expectancy. The primary objective of the study was to assess the proportion of patients free from progression at 2 months from the beginning of the treatment. Secondary endpoints were a series of pharmacodynamic and pharmacokinetic analyses such as the investigation of the pharmacokinetic parameters of UFT and its metabolites and the modulation of CD133 gene expression, vascular endothelial growth factor (VEGF), soluble VE-cadherin (sVE-C) and thrombospondin-1 (TSP-1) plasma levels as possible pharmacokinetic/pharmacodynamic markers of the therapy.

## Patients and methods

### Patient selection

Main eligibility criteria included: (1) histologically confirmed diagnosis of colorectal or other gastrointestinal adenocarcinoma with metastatic disease; (2) previous chemotherapy with fluoropyrimidines, oxaliplatin, irinotecan, when indicated; (3) measurable disease progressing during or within 3 months from the end of the treatments; (4) life expectancy greater than 3 months; (5) Eastern Cooperative Oncology Group performance status ≤ 2; (6) adequate bone marrow, renal and liver function (leukocytes ≥3,000 mm^−3^, platelet ≥100,000 mm^−3^, creatinine ≤2 mg/dL^−1^, total bilirubin ≤ 1.5x institutional upper limit of normal, AST/ALT ≤ 5x institutional upper limit of normal). Study exclusion criteria were as follows: brain metastasis, symptomatic cardiac disease, recent myocardial infarction, active infections and inflammatory bowel disease.

### Treatment schedule and doses

Patients received on day 1 a single administration of CTX 500 mg/m^2^ as i.v. bolus and, from day 2, 50 mg CTX p.o. once daily plus 100 mg UFT p.o. and 200 mg CXB p.o. twice a day. From day 2, the treatment was continued without interruption until either disease progression, unacceptable toxicities, deterioration of performance status, or patient refusal to continue treatment. No dose reduction for toxicities was applied. To prevent nausea and vomiting, metoclopramide 10 mg i.v plus dexamethasone 4 mg i.v. were administered before CTX i.v. chemotherapy on day 1. Loperamide 2 mg orally every 2 h and oral rehydration were prescribed in the event of delayed diarrhea. No prophylactic treatment with supportive cytokines such as G-CSF was recommended.

### Clinical assessment, toxicity and response criteria

Pretreatment evaluation included complete history and physical examination, performance status assessment, complete blood count and differential, platelet count, complete blood profile, tumor markers, urinalysis, ECG, chest X-ray or computed tomography scan, abdominal computed tomography scan and/or sonogram, and any other appropriate diagnostic procedure to evaluate metastatic sites. During treatment, a physical examination, a complete blood cell count, blood profile, urinalysis and toxicity evaluation were performed every 3 weeks. Sites of metastatic disease were radiologically re-evaluated every 2 months, according to the RECIST criteria [21]. A chest X-ray and/or an abdominal sonogram were repeated at least every 6 months if there was no evidence of lung or abdominal disease, respectively. Toxicities were scored according to the standard NCI Common Terminology Criteria for Adverse Events (version 3.0). Duration of responses was calculated from the first day of treatment to the date of first observation of progressive disease or last examination.

### Pharmacokinetics of tegafur, 5-FU, 5-fluoro-5,6-dihydrouracil (5-FUH_2_), GHB and uracil

The pharmacokinetic analysis of tegafur, 5-FU, 5-FUH_2_, GHB and uracil were performed as previously described [22–24] with minor modifications. Blood samples (4 ml each) for pharmacokinetic assays were taken from an indwelling i.v. cannula placed in an antecubital vein at baseline, 30 min, 1, 1.5, 2, 3 and 5 h at day 1, 28 and 56 after the beginning of UFT oral administration. Blood was centrifuged (5 min, 4,000 rpm, 4°C) to separate plasma, which was stored at −80°C and assayed within 1 week. The simultaneous assay of 5-FU and 5-FUH_2_ in human plasma was performed by a validated, nonradioactive reverse-phase HPLC method with ultraviolet detection. Briefly, 0.5 ml of plasma, mixed with sodium acetate and sodium sulfate, were extracted with 7 ml of n-propyl alcohol/diethyl ether. Samples were centrifuged to separate the organic phase, which was evaporated to dryness; they were then reconstituted with 250 μl of mobile phase (50 mmol/l potassium phosphate; pH 4.0) and finally injected into the LC Module I Plus HPLC with an ultraviolet detector set at 215 nm (Waters, Milford, USA). 5-FU and 5-FUH_2_ were separated on Hypersil BDS C18 stationary phase (Alltech, Deerfield, USA), eluted with 1 ml/min of mobile phase. The data analysis was performed by use of Millenium 2.1 software (Waters). Standard calibration curves were obtained by adding 5-FU and 5-FUH_2_ to 0.5 ml of blank plasma obtained from healthy donors on each day of analysis, resulting in final concentrations that ranged from 0.08 to 75 μg/ml. For the analysis of tegafur (FT) and uracil (U), plasma samples (1.0 ml) were adjusted with 0.1 ml of 0.5 M NaH_2_PO_4_ buffer and 8 ml ethyl acetate were added. After extraction and centrifugation, the organic layer was removed and evaporated under N_2_ at 50°C. The residue was dissolved in 50 μl of methanol, and 20 μl were injected into the HPLC with an ultraviolet detector set at 270 nm (Waters, Milford, USA). FT and U were separated on Hypersil BDS C18 stationary phase (Alltech, Deerfield, USA), eluted with 1 ml/min of mobile phase. The data analysis was performed by use of Millenium 2.1 software (Waters). Mobile phase was 0.01 M sodium acetate buffer (pH 4):methanol (85:15, v/v) as eluent. The retention times were 6.4, 2.7 min for FT and U, respectively. Standard calibration curves were obtained by adding FT and U to 0.5 ml of blank plasma obtained from healthy donors on each day of analysis. Sensitivity limit of quantitative analysis in plasma was 0.1 μg/ml. In order to detect GHB, 200 μl of plasma were treated with 500 μl of acetonitrile, using α -hydroxy-isovaleric acid (200 ng/ml) as internal standard. After agitation and centrifugation (9,000*g* for 10 min), the supernatant was collected and evaporated to dryness under nitrogen flow. The residue was derivatized by adding 50 μl N,O-bis(trimethylsilyl)trifluoroacetamide + 1% trimethylchlorosilane (BSTFA + 1% TMCS), then incubated for 30 min at 70°C. An aliquot (1 ml) of the derivatized extract was directly injected into GC/MS using a TRACE gas chromatograph equipped with a Polaris Q as mass detector and an AS2000 as autosampler (Thermo Finnigan, Rodano, Italy). The flow of carrier gas (helium, purity grade N55) through the column (Restek, Palo Alto, USA; Rtx-5MS capillary column, 30 m × 0.25 mm × 0.25 μm film thickness) was 1.0 ml/min. The injector temperature was 280°C and splitless injection was employed with a split valve off-time of 1.0 min. The column oven temperature was programmed to rise from an initial temperature of 65°C, maintained for 1 min, to 140°C at 22°C/min, then 140°C for 3 min, then to 290°C at 50°C/min and maintained at 290°C for the final 5 min. Data were recorded in full scan and ions monitored were: *m/z* *233*, 73 and 147 and *m/z* 73, *145* and 219 for GHB and α-hydroxy-isovaleric acid, respectively (the underlined ions were used for quantitation).

Individual plasma concentration profiles of tegafur and its catabolites were fitted according to a two-compartment model by use of nonlinear least squares regression analysis (MwPharm software, version 3.60; MediWare, Groningen, The Netherlands). The area under the curve (AUC) of tegafur, 5-FU, 5-FUH_2_, GHB and uracil was calculated by the trapezoidal method for the area from time 0 to the time of the last measurable concentration. The maximum plasma concentration (C_max_) and time to reach C_max_ (T_max_) were identified from the inspection of tegafur and its catabolite concentration–time plots.

### CD133 gene expression by real time RT-PCR in peripheral blood mononuclear cells (PBMCs)

Before drug administration and at day 28, 56, 84 and 112, 10 ml of blood were drawn from the antecubital vein of patients. PBMCs were collected as previously published [7]; the resulting pellet was immediately frozen in liquid nitrogen and stored at −80°C. As previously described [25], RNA was reverse transcribed and the resulting cDNA was diluted and then amplified by QRT-PCR with the Applied Biosystems 7900HT sequence detection system. CD133 validated primer were purchased from Applied Biosystems (Assay ID Hs00195682_m1). The PCR thermal cycling conditions and optimisation of primer concentrations were followed as *per* manufacturer’s instructions. Amplifications were normalized to GAPDH and the quantitation of gene expression was performed using the ΔΔ*C*_*t*_ calculation; the amount of CD133, normalized to the endogenous control and relative to the calibrator (PBMC sample at day 0), is given as $2^{{ - \Updelta \Updelta C_{t} }}$. The data are presented as the percentage of $2^{{ - \Updelta \Updelta C_{t} }}$ at day 0 (before the beginning of metronomic schedule).

### Plasma VEGF, TSP-1 and sVE-C levels detection by ELISA

Plasma samples obtained at the same days of PBMC collection were assessed for VEGF, TSP-1 and sVE-C levels using commercially available ELISA kits. Each sample was assayed for human VEGF and TSP-1 concentrations by the ELISA Kit Quantikine^®^ (DVE00 and DTSP10, R&D Systems, Minneapolis, MN, USA) and for soluble VE-cadherin by Instant ELISA Kit (Bender Medsystems, Wien, Austria). Measurements were performed by the microplate reader Multiskan Spectrum (Thermo Labsystems, Milan, Italy) set to 450 nm (with a wavelength correction set to 540 nm).

### Statistical analysis

The primary objective of the study was to evaluate the percentage of patients not progressed within 2 months from the beginning of metronomic CTX plus UFT and celecoxib regimen. In phase II studies of chemotherapy administered for palliation in patients with mCRC or with gastrointestinal tumors, already treated with standard chemotherapy treatments, a rate of approximately 20% of patients free from progression within 2 months of treatment was generally observed. Our study of metronomic UFT plus CTX and CXB aimed to achieve an increase from 20 to 40% in the proportion of patients not progressed at 2 months from starting treatment.

According with the single-stage design described by Fleming and A’Hern, choosing a parameter P0 (percentage of patients free from progression at 2 months: null hypothesis) = 0.20, and P1 (proportion of patients free from progression at 2 months: alternative hypothesis) = 0.40, and considering the errors α and β of 0.10 and 0.10, the study required the enrollment of at least 36 evaluable patients. Study treatment was considered promising when at least 11 patients were progression free at 2 months. Progression free survival (PFS) and overall survival (OS) were calculated from the date of progression or death/loss to follow-up, respectively, using the Kaplan–Meier method. The log-rank test was used to compare survival between patients having stable disease (SD) and progressive disease (PD). Statistical analysis by ANOVA, followed by the Student–Newman–Keuls test, was used to assess the statistical differences of pharmacokinetic and pharmacodynamic data. Correlations between pharmacokinetic parameters were investigated by linear regression analysis. Cut off values for 5-FU C_max_ and AUC parameters were found with nonparametric receiver operating characteristic (ROC) analysis, performed to assess the accuracy of pharmacokinetic parameters to discriminate between stable and progressive disease groups of patient. Statistical analyses were performed using the GraphPad Prism software version 5.0 (GraphPad Software Inc., La Jolla, CA, USA).

## Results

### Patients and toxicity

As outlined in Table 1, mainly patients with advanced metastatic colorectal cancer (mCRC; *n* = 30) were entered into the study. The final analysis was conducted on the total number of 38 patients. Median age was 71 years (range, 51–87 years), ECOG performance status was 0–1 in 37 patients and 2 in one. As reported, the entire study population was heavily pretreated and in particular oxaliplatin- and fluoropyrimidines-based chemotherapy was administered to all patients with mCRC. Of note, 36 and 20% of patients with mCRC received in addition cetuximab and bevacizumab, respectively. Patients with mCRC received a median number of three treatments (range 2–5) before entering the study. The patient with gastric cancer received a first-line including oxaliplatin and 5-fluorouracil and the remainder of the patients received at least a chemotherapy treatment including gemcitabine. The patients with gastrointestinal cancers received a median number of treatments of two. A median of 12 weeks per patient (range, 2–63 weeks) of therapy were administered using metronomic schedule. Cessation of treatment was due to disease progression in all patients.

**Table 1:** Patient characteristics

*Number of patients*	38
Median age (range)	71 years (51–87)
Gender (*male/female*)	25 (66%)/13 (34%)
PS ECOG (*0/1/2*)	13 (34%)/24 (63%)/1 (3%)
*Primary tumor sites n* (*%*)	
Colon-rectum	30 (79%)
Gastric	1 (3%)
Pancreas	2 (5%)
HCC	2 (5%)
Biliary tract	3 (8%)
*Metastatic sites n* (*%*)	
Liver	26 (68%)
Lung	24 (63%)
Lymph node	14 (37%)
Bone	4 (10%)
Peritoneum	6 (16%)
Others	9 (24%)
*No. of metastatic sites* (*%*)	
Single	7 (19%)
Multiple	31 (81%)
*No. of previous cancer treatments for mCRC median* (*range*)	3 (2–5)
*Drugs previously used for the treatment of mCRC*	
Oxaliplatin	30 (100%)
Irinotecan	27 (90%)
Fluoropyrimidine	30 (100%)
Cetuximab	11 (36%)
Bevacizumab	6 (20%)

*PS ECOG* performance status Eastern Cooperative Oncology Group, *mCRC* metastatic colorectal cancer

All patients were assessable for toxicities, which were very uncommon. In particular, we did not observe any toxicities higher than grade 1. Four (10.5%) and six patients (15.7%) experienced, respectively, a transient grade 1 diarrhea and nausea, which resolved without interrupting the treatment. No notable hematological toxicities were observed.

### Antitumor activity and survival

**Fig. 1:**
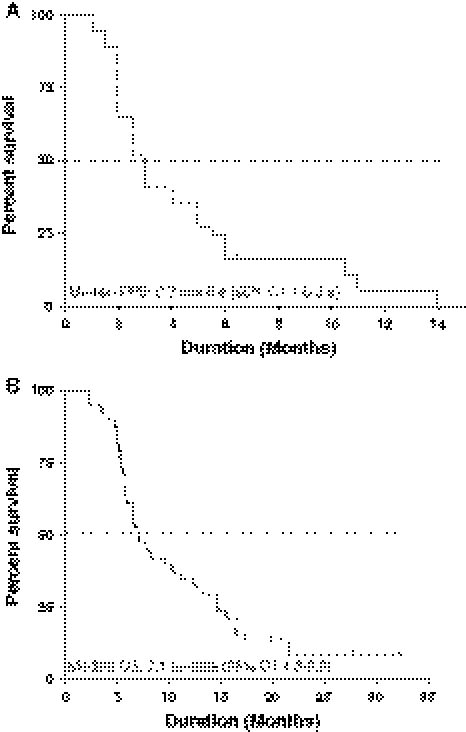
Progression free survival (**a**) and overall survival (**b**) curves calculated by the Kaplan–Meier method from the first day of the metronomic CTX, UFT and CXB schedule

All patients had at least a measurable lesion according to Response Evaluation Criteria in Solid Tumors (RECIST) criteria [21]. Of 38 patients assessable for response, we observed 17 patients (45%) with stable disease (SD), that lasted a median period of 5.8 months (range, 2.5–14.6 months), and 21 patients (55%) with progression of disease (PD) at the first clinical evaluation. Among mCRC patients, thirteen (43%) obtained a stabilization of disease that lasted a median period of 5.1 months (range, 2.8–14.1 months) with an observed median overall survival in the responders of 12.1 months (range, 5–14 months). The median duration of SD response in the remaining four non-CRC patients (2 patients with pancreatic cancer, one patient with cancer of the biliary tract and one patient with hepatocellular carcinoma) was 5.6 months (range, 2.4–8.9 months). After a median follow-up of 18.3 months, for the entire population median progression free survival and median overall survival were 2.7 months (95% CI, 1.6–3.9 month; Fig. 1a) and 7.1 months (95% CI, 4.3–9.9 months; Fig. 1b), respectively.

### Pharmacokinetics

**Fig. 2:**
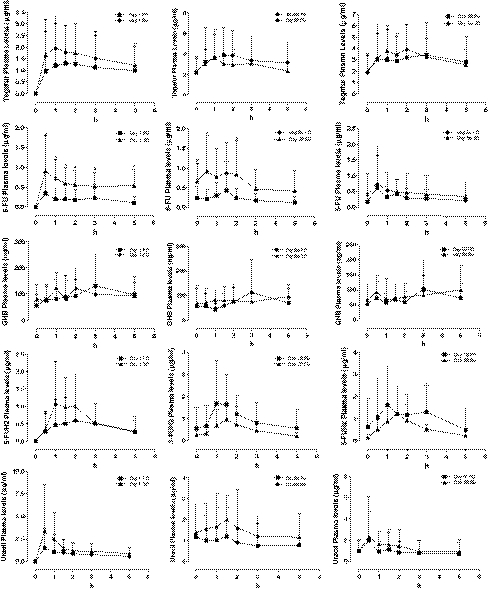
Plasma levels of Tegafur, 5-fluorouracil (5-FU), 5-fluoro-5,6-dihydrouracil (5-FUH2), Uracil and gamma-hydroxybutyric acid (GHB) in 13 stable disease (SD) patients and 14 progressive disease (PD) patients at day 1, 28 and 56, receiving the metronomic CTX, UFT and CXB schedule. *Points* mean; *bars* Standard Deviation. *P < 0.05 PD versus SD; ^#^ *P* < 0.01 PD versus SD

The pharmacokinetic analyses of tegafur, 5-FU, 5-FUH_2_, uracil and GHB were performed in 27 patients (21 patients with colon cancer, 2 patients with pancreatic cancer, 2 patients with cancer of the biliary tract and 2 patients with hepatocellular carcinoma). A statistically significant difference in the values of area under curve (AUC) and maximum plasma concentration (C_max_) on day 1 compared to day 28 and day 56 of tegafur was found (data not shown). Among the numerous data obtained by the comparison of the pharmacokinetic parameters of PD (*n* = 14) and SD (*n* = 13) patients, statistically significant differences were demonstrated in 5-FU AUC values both on day 1 and day 28 (data not shown). Moreover, the difference of 5-FU C_max_ values on day 1 between the group of patients in PD and SD patients was found to be statistically significant (Fig. 2). The analysis of 27 patients, after the first intake of 100 mg UFT tablet, revealed a significant difference between the PD and SD group at day 1 for the 5-FU AUC (0.997 ± 1.271 vs. 2.765 ± 1.709 h × μg/ml, respectively, *P* < 0.05; and C_max_ (0.453 ± 0.573 vs. 1.134 ± 0.749 μg/ml, respectively, *P* < 0.05).

**Fig. 3:**
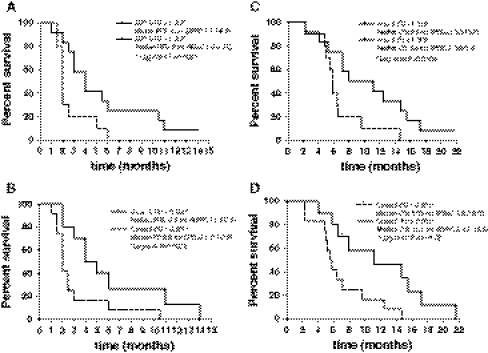
PFS according to 5-FU AUC (**a**) and C_max_ (**b**) cutoff values obtained by a ROC analysis (see “Results” section of the text) and OS according to 5-FU AUC (**c**) and C_max_ (**d**) values at day 1 of treatment of patients administered with the metronomic CTX, UFT and CXB schedule

Cut off values for 5-FU AUC and 5-FU C_max_ parameters higher than 1.313 h × μg/ml and 0.501 μg/ml, respectively, could predict the clinical stabilization of the disease at day 1 with a sensitivity of 81.82% and a specificity of 69.23 and 76.92%, respectively. Even more interesting, patients with the 5-FU AUC and C_max_ pharmacokinetic parameters at day 1 greater than 1.313 h × μg/ml and 0.501 μg/ml, respectively, showed a significant prolonged PFS (Fig. 3a, b) and a significant increase of the OS (Fig. 3c, d).

### Pharmacodynamics

**Fig. 4:**
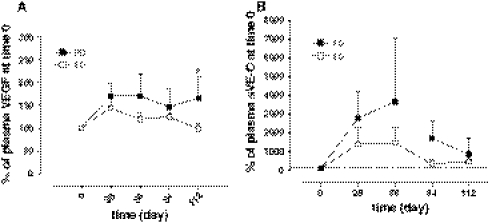
VEGF (**a**) and sVE-C (**b**) plasma level profiles of patients (*n* = 35) administered with the metronomic CTX, UFT and CXB schedule. *Points* mean; *bars* SD. The data are presented as percentage of the concentration at day 0 (before the beginning of the metronomic treatment) of each individual patient. *P < 0.05 PD versus SD. *PD* progressive disease, *SD* stable disease

**Fig. 5:**
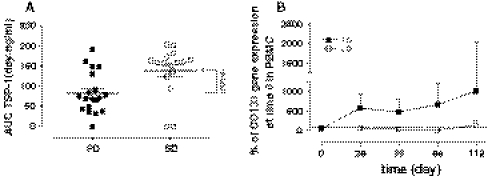
TSP-1 AUCs of patients (*n* = 35) administered with the metronomic CTX, UFT and CXB schedule (**a**). Mean ± SD; *P < 0.01 progressive disease (PD) versus stable disease (SD). CD133 gene expression in PBMC of patients administered with the metronomic CTX, UFT and CXB schedule (**b**). *Points* mean, *bars* SD. The data are presented as percentage of $2^{{ - \Updelta \Updelta C_{t} }}$ at day 0 (before the beginning of the metronomic treatment) of each single patient

The pharmacodynamic analyses were performed in 35 patients. After starting the metronomic schedule, the VEGF-A and sVE-C plasma levels remained markedly greater (but not statistically significant) in the PD (*n* = 19) group of patients when compared to the SD (*n* = 16) group of patients (Fig. 4a, b, respectively). Interestingly, the measurement plasma levels of endogenous TSP-1 during the time (measured by the TSP-1 AUC) was significantly higher in SD than PD patients (Fig. 5a). After the beginning of the metronomic treatment, a lower CD133 gene expression was consistently maintained in the SD patients and resulted similar to the baseline observation. Patients with progressive disease showed a substantial increase of CD133 gene expression after 4 weeks from the beginning of treatment, maintaining these high levels for at least 4 months (Fig. 5b).

## Discussion

The primary objective of the study was to assess whether a metronomic chemotherapy regimen including UFT plus CTX and CXB could increase the progression-free survival at 2 months in a population of metastatic gastrointestinal cancer patients already treated with several chemotherapy lines. The observed results showed that more than 40% of patients were free of disease progression at 2 months from the beginning of the treatment with a median progression free survival and a median overall survival, respectively of 2.7 and 7.1 months. The results seem suggest a possible antitumor activity of this metronomic regimen, comparable with those observed in the same setting of patients treated with other third/fourth lines of chemotherapy.

Previous clinical experiences have showed that subsequent treatments to a first–second line of chemotherapy, generally still including a fluoropyrimidine, in this setting of refractory patients, produce a poor response rate (generally less than 10%), with a low median progression free and a median overall survival, generally around a few months [26]. Furthermore, a high percentage of patients were reported to experience severe toxicity (≥grade 3 of NCI scale), further limiting the use of these regimens [27, 28]. Our results confirm, instead, the previous published reports and experience regarding the generally excellent tolerability profile of metronomic chemotherapy, which basically does not show treatment-related toxicity greater than grade I–II according to the NCI scale, as previously shown in cancer patients with advanced colorectal cancer [7], prostate cancer [29, 30], breast cancer [31–33], and gastrointestinal cancer [10]. Apart from the well-known low toxicity of metronomic chemotherapy, another possible explanation for the good tolerability of our schedule could be found in the co-administration of celecoxib. Indeed, Lin EH et al. [34] have shown a potential benefit for the toxicity profile of capecitabine if this fluoropyrimidine was associated with celecoxib. Celecoxib may improve clinical outcomes and reduce toxicities (e.g. the gastrointestinal ones) when administered in association with a 5-FU prodrug such as UFT.

With particular reference to mCRC patients, who represented the 79% of the whole study population (30 patients), although no complete or partial responses were observed, the metronomic UFT/CTX and CXB combination produced a stable disease in the 43% of patients that lasted a median period of 5.1 months, with an observed median overall survival in the responders of 12.1 months (range, 5–14 months). These preliminary results, for the first time, suggest a possible role of this metronomic regimen in a population of treated refractory mCRC patients.

No previous clinical experiences have been reported for a metronomic regimen including UFT/CTX and CXB in mCRC patients and, in general, metronomic chemotherapy has been poorly evaluated in this setting. A few papers are currently published in the scientific literature. Young et al. [10] evaluated the impact of a combination metronomic treatment with CTX, vinblastine and rofecoxib in patients with advanced tumours, including only 13 with mCRC patients, observing a partial response in one patient and a stable disease in other patients with a progression free survival of 12 and 7 months, respectively. Moreover, in a recent phase II randomized study of 88 patients with different cancer types, 11 mCRC patients were included and treated with CTX metronomic, showing only disease progressions [35].

In light of these results, this minimally toxic metronomic regimen with UFT, CTX and CXB could represent a possible therapeutic option for patients with mCRC who have failed chemotherapies with or without target therapies. Furthermore, remarkable results are represented by pharmacokinetic and pharmacodynamic data that show a possible correlation between these laboratory analyses and antitumor activity of the treatment. Various tegafur-based schedules have been evaluated in a large number of phase II and III studies; the investigated doses varied from 300 to 600 mg/m^2^/day [36]. A standard UFT schedule have been published by Shirao et al. [37] in advanced colorectal cancer with a recommended dose of tegafur 300 mg/m^2^ daily, combined with leucovorin 75 mg/day for 28 days with subsequent courses repeated after 7-days intervals. Metronomic chemotherapy is a frequent (even daily), prolonged low dose administration of a chemotherapeutic drug, thus we have decided to design our study based on the prolonged oral administration of tegafur at a fixed dose of 100 mg twice a day which is approximately one third of the published, standard, daily total dose.

A strong rationale of a metronomic chemotherapy based on the association between UFT and CTX derives directly from their synergistic antitumor activity in experimental mouse models of metastatic breast and hepatocellular carcinoma [19, 38]. The clinical combination of UFT and CTX has also been evaluated in women with metastatic breast cancer, although not at a metronomic dosing, by Ogawa et al. [39] who found that daily treatment with UFT (300–400 mg) and CTX (100–150 mg), both given orally, was associated with a 35% response rate. A relevant finding of our study are related to the possible use, in future prospective clinical studies, of UFT pharmacokinetic parameters at the very first intake of the drug in order to predict the efficacy and the PFS and OS of our patients. Indeed, the pharmacokinetic analysis of 27 patients after the first intake of UFT revealed a significant difference between the PD and SD groups at day 1 in 5-FU (the main tegafur active metabolite) AUC and C_max_. The data obtained by means of ROC analysis on both parameters may suggest that patients with 5-FU AUC and C_max_ higher than 1.313 h × μg/ml and 0.501 μg/ml have greater clinical benefit from metronomic chemotherapy accompanied by a prolonged PFS and OS. Although promising, further studies should be performed in order to validate our preliminary findings. Indeed, a perfect separation between PD and SD groups by ROC analysis is rare, as also reported by Zweig and Campbell [40], because the distribution of the test results tend to overlap. Despite the limitation of this analysis, our results are the first attempt to identify a pharmacokinetic cut-off value in a clinically relevant population. Interestingly, the found 5-FU concentrations are far less from those that could be achieved by standard 5-FU chemotherapeutic schedules [41] which primarily target tumor cells. Moreover, in patients who are heavily pre-treated, and whose tumor are resistant to fluoropyrimidines, the concentrations detected would not be expected to exert a direct cytotoxic effect. Indeed, it is plausible to suggest a different mechanism of action of metronomic UFT, perhaps more related to the antiangiogenic effect on proliferating endothelial cells or circulating endothelial precursors (CEPs) caused by low concentration of 5-FU. The anti-angiogenic effects of UFT are amplified when administered at lower, non toxic daily doses [19]. Preclinical studies have demonstrated that GHB and gamma-butyrolactone (GBL), active metabolites of UFT, are involved in the expression of anti-angiogenic activity of UFT [42, 43]. In particular, in vitro studies have shown that GHB inhibits endothelial cells with IC_50_ values of 25.8 ng/ml. In our experimental setting, the mean plasma GHB C_max_ varied during the treatments from 161 to 127 ng/ml. These concentrations are consistent with previous published experience [44] and, above all, with a direct antiangiogenic activity of GHB that could synergize with low 5-FU concentrations.

The hypothesis that our metronomic UFT/CTX and CXB combination schedule could be active through a marked antiangiogenic activity is also supported by the pharmacodynamic markers we investigated. Indeed, using a quantitative reverse transcription-PCR approach for CD133 RNA evaluation, we have found that this marker increased during the treatment only in the PD group of patients. CD133/prominin-1 is expressed on several primitive cells such as hematopoietic stem and progenitor cells derived from bone marrow, fetal liver and peripheral blood, and developing epithelium, including circulating endothelial progenitor cells (CEPs) [45]. Moreover, previously published studies have suggested the role of this protein as a marker of cancer stem cells in metastatic colorectal cancer [46, 47]. Lower expression of CD133 in SD patients might be linked also to a reduced CEP mobilization caused by metronomic chemotherapy [48, 49], and consequently to a better response to the therapy as CD133-positive cells are associated with chemoresistance [50]. Although it is not possible to ascribe with certainty the found CD133 expression in PBMC to CEPs or to other progenitor cells, our findings may cautiously represent a possible future molecular biomarker of metronomic chemotherapy. Moreover, VE-C, a protein involved in the process of tumor vascularization [51] and associated with bone marrow–derived CEPs [52], showed lower variation of plasma concentrations in SD patients when compared to PD patients, suggesting a possible relationship between circulating VE-C levels and activity of the metronomic protocol. Indeed, a lower increase of VE-C gene expression was also demonstrated during the treatment with a cyclophosphamide-based metronomic schedule in responder patients with metastatic prostate cancer [30]. Finally, we have found that SD patients showed a significant higher exposure to the endogenous antiangiogenic factor TSP-1 during treatment with lower plasma VEGF levels when compared with the PD patients. These results are consistent with the reported upregulation of TSP-1 as one of the mechanisms of action of metronomic low-dose chemotherapy regimens [53].

In conclusion, our results show that metronomic UFT/CTX chemotherapy with CXB is feasible, well tolerated and associated with a promising antitumor activity in heavily pretreated gastrointestinal cancer patients. Significant differences in pharmacokinetic parameters were found between SD and PD patients suggesting the presence of potentially promising predictive markers for further studies (disease-oriented) to determine optimal UFT doses at the very beginning of the treatment, thus improving the patient’s benefit, including survival.
